# An add-on training program involving breathing exercises, cold exposure, and meditation attenuates inflammation and disease activity in axial spondyloarthritis – A proof of concept trial

**DOI:** 10.1371/journal.pone.0225749

**Published:** 2019-12-02

**Authors:** G. A. Buijze, H. M. Y. De Jong, M. Kox, M. G. van de Sande, D. Van Schaardenburg, R. M. Van Vugt, C. D. Popa, P. Pickkers, D. L. P. Baeten

**Affiliations:** 1 Department of Orthopaedic Surgery, Amsterdam UMC, University of Amsterdam, Amsterdam, The Netherlands; 2 Department of Rheumatology and Clinical Immunology, Amsterdam UMC, University of Amsterdam, Amsterdam Rheumatology and Immunology Center, Amsterdam, The Netherlands; 3 Department of Intensive Care Medicine, Nijmegen Institute for Infection, Inflammation and Immunity, RadboudUMC Nijmegen, Nijmegen, The Netherlands; 4 Reade, Amsterdam Rheumatology and Immunology Center, Amsterdam, The Netherlands; 5 Department of Rheumatology and Clinical Immunology, Amsterdam UMC, Vrije Universiteit Amsterdam, Amsterdam, The Netherlands; 6 Department of Rheumatology, Sint Maartenskliniek, Nijmegen, The Netherlands; 7 Department of Rheumatology, RadboudUMC Nijmegen, Nijmegen, The Netherlands; University of Bern, SWITZERLAND

## Abstract

**Objectives:**

The primary objective of this trial was to assess safety and anti-inflammatory effects of an add-on training program involving breathing exercises, cold exposure, and meditation in patients with axial spondyloarthritis

**Methods:**

This study was an open-label, randomised, one-way crossover clinical proof-of-concept trial. Twenty-four patients with moderately active axial spondyloarthritis(ASDAS >2.1) and hs-CRP ≥5mg/L were included and randomised to an intervention (n = 13) and control group (n = 11) group that additionally received the intervention after the control period. The intervention period lasted for 8 weeks. The primary endpoint was safety, secondary endpoints were change in hs-CRP, serum calprotectin levels and ESR over the 8-week period. Exploratory endpoints included disease activity measured by ASDAS-CRP and BASDAI, quality of life (SF-36, EQ-5D, EQ-5D VAS), and hospital anxiety and depression (HADS).

**Results:**

We found no significant differences in adverse events between groups, with one serious adverse event occurring 8 weeks after end of the intervention and judged ‘unrelated’. During the 8-week intervention period, there was a significant decline of ESR from (median [interquartile range] to 16 [9–26.5] to 9 [5–23] mm/hr, p = 0.040, whereas no effect was found in the control group (from 14 [8.3–27.3] to 16 [5–37] m/hr, p = 0.406). ASDAS-CRP declined from 3.1 [2.5–3.6] to 2.3 [1.9–3.2] in the intervention group (p = 0.044). A similar trend was observed for serum calprotectin (p = 0.064 in the intervention group versus p = 0.182 in the control group), but not for hs-CRP.

**Conclusions:**

This proof-of-concept study in axial spondyloarthritis met its primary endpoint with no safety signals during the intervention. There was a significant decrease in ESR levels and ASDAS-CRP upon the add-on training program in the intervention group. These findings warrant full-scale randomised controlled trials of this novel therapeutic approach in patients with inflammatory conditions.

**Trial registration:**

ClinicalTrials.gov; NCT02744014

## Introduction

Previous research in healthy individuals exposed to experimental endotoxemia showed that the innate immune response can be voluntarily modulated by a training program involving breathing exercises, exposure to cold and meditation (further referred to as: ‘add-on training program’).[1,2] Practicing the techniques learned in the add-on training program induced intermittent respiratory alkalosis and hypoxia, as well as profoundly increased plasma epinephrine levels, indicating activation of the sympathetic nervous system. These changes correlated with increased plasma levels of the anti-inflammatory cytokine IL-10 and attenuated levels of pro-inflammatory mediators such as TNF-α, IL-6, and IL-8 during experimental endotoxemia.[2]

The study of Kox et al[2] evaluated short term effects of this add-on training program in a controlled experimental model of acute inflammation in healthy individuals. It is unknown whether the same intervention could potentially lead to suppression of inflammation in patients with chronic inflammatory diseases. And, more importantly, it is not known whether this training program can safely be applied in patients with a chronic inflammatory disorder.

We designed a proof of concept (PoC) trial aimed to assess whether this well-defined add-on training program could modulate innate immune responses in a prototypical chronic inflammatory disease. We selected axial spondyloarthritis (axSpA)[3] as model disease since this chronic rheumatic inflammation of the spine a) involves altered innate immune responses,[4, 5] b) affects mainly young adults with few comorbidities and concomitant medication, allowing for an unbiased efficacy and safety assessment, and c) persists often for years as stable mild-to-moderate disease. Despite recent advances in therapeutic options for axSpA it is still not possible to sufficiently control disease activity in all patients, as only 60–70% of the patients respond to treatment, of whom 30% only partially. Remission is only achieved in 20% of the patients. This indicates a clear opportunity for additional treatment options, such as the add-on training program, to improve the outcome in these patients.

This study addresses the following primary research question: Can this add-on training program safely be applied in patients with active axial spondyloarthritis? C- reactive protein (CRP), erythrocyte sedimentation rate (ESR) and calprotectin levels are evaluated as secondary biological endpoints to investigate potential impact on inflammatory response. The exploratory outcomes are other inflammatory markers and the patient-reported outcomes ASDAS-CRP, Bath Ankylosing Spondylitis Disease Activity Index (BASDAI), quality of life measures (SF-36, EQ-5D, EQ-5D VAS), and hospital anxiety and depression (HADS). As this is the first study to investigate the safety and efficacy of this intervention in patients with a chronic inflammatory disease, we did not attempt to decipher the mechanism of action of the add-on training program.

## Methods

### Study design

The study design used for this proof-of-concept trial was an open-label randomised one-way crossover design to rule out regression to the mean (S1 Fig). This intervention is not suitable to compare to a genuine placebo effect. A concealed computer-based system randomised participants to an early intervention group or late intervention group in a 1:1 ratio. Stratification was performed for duration of disease and disease activity by ASDAS-CRP.

The early intervention group started with the intervention at baseline and continued with the intervention until week 8. Study visits in the intervention period were done at baseline, week 4 and week 8. Sixteen weeks after the intervention period, thus at week 24, a follow-up visit was done. The total study duration for the early intervention group was 24 weeks, in which 4 study visits were done.

The late intervention group started with the control period that lasted for 8 weeks. Study visits were performed at baseline, week 4 and week 8. After 8 weeks, the late intervention group started with the intervention and continued with the intervention for 8 weeks. The week 8 visit of the control period also served as the baseline visit for the intervention period. Study visits in the intervention period for the late control group were done after 4 and 8 weeks of intervention (thus at week 12 and week 16). Sixteen weeks after the intervention period, thus at week 32, a follow-up visit was done. The total study duration for the late intervention group was 32 weeks, in which 6 study visits were done.

Data from the first 8 weeks of study participation of the late intervention group were used as control data. Data from both intervention periods (of the early and late intervention group) were combined in the analysis. In both groups, the 8-week intervention period was followed by a 16-week safety follow-up period. Patients were enrolled between May and December 2016. Participating centres were the Academic Medical Centre (AMC) and Bernhoven Hospital. All training sessions took place in the AMC.

### Patients

Patients aged between 18 and 55 years were eligible if they had a clinical diagnosis of axSpA according to the treating physician, fulfilled the ASAS classification criteria[6] and had active disease defined as ASDAS>2.1[7] and a high-sensitive CRP (hs-CRP) ≥5mg/L. Patients were allowed to use concomitant non-steroidal anti-inflammatory drugs (NSAIDs), corticosteroids (prednisone equivalent up to 10mg/day) and disease modifying anti-rheumatic drugs (DMARDs) (both synthetic and biologic) provided they have been initiated at least 8 weeks before screening (exception: two weeks for NSAIDs) and that the dose had been stable for at least 6 weeks prior to screening. Doses also had to remain stable throughout the study (from screening to end of intervention at week 8). Exclusion criteria were significant comorbidities that, in the opinion of the investigators, could interfere with the study or lead to deleterious effects for the patient, a recent history or persistence of an infection requiring hospitalization or antibiotic treatment within 4 weeks of baseline and pregnancy.

All participants gave written informed consent. Patients received a travel allowance that was dependent on the distance of the patients’ residence to the training site, but no other reimbursement. This study was approved by the Medical Ethics Committee of the Academic Medical Centre in Amsterdam under reference number 2015_328.

### Intervention

The intervention consisted of an 8-weeks add-on training program comprising three elements: 1) breathing exercises (further detailed below), 2) gradual cold exposure (immersions in ice cold water), and 3) meditation (third eye meditation).

The breathing techniques consisted of two exercises. First, patients were asked to hyperventilate for an average of 30 breaths. Subsequently, the patient exhaled and held their breath in an unforced manor for ∼2–3 min until they felt a stimulus to inhale (“retention phase”). The duration of breath retention was entirely at the discretion of the patient himself. For safety reasons, it was instructed to not hold the breath longer than 3.5 minutes. Breath retention was followed by a deep inhalation breath, which was held for 10 s. Subsequently a new cycle of hyper/hypoventilation began. After the last cycle, patients were instructed to do a strenuous exercise such as push-ups. The induced state of intermittent respiratory alkalosis and hypoxia typically “empowers” the patient to outperform their standard capability in any physical exercise. The second breathing exercise consisted of deep inhalations and exhalations in which every inhalation and exhalation was followed by breath holding for 10 s, during which the patient tightened all his body muscles. An additional element this part of the training program consisted of strength exercises (e.g., push-ups and yoga balance techniques).

During the intervention period, patients voluntarily exposed themselves to cold in two ways. During weekly training sessions, the patients immersed whole-body in ice-cold water (0–1°C) for several minutes, incrementally up to a maximum of 5 minutes (for safety reasons). At home, daily cold showers were taken incrementally up to 5 minutes (10–14°C).

The so-called “third eye meditation,” is a form of meditation including visualizations aimed at total relaxation. It consisted of an unguided meditation (in silence) with the eyes closed in any posture as desired by the patient for a period of 15–20 minutes. It was generally used at the end of each training session.

Consistent with the previous study,[2] patients were trained at the academic rehabilitation centre by a Dutch individual Wim Hof and four trainers who previously received an instructor course by Wim Hof to become a trainer. Medical personnel were present during all training sessions. During the first 4 weeks, participants had group trainings twice weekly, the second 4 weeks once weekly. During the intervention period, and after extensive written and oral instructions, participants practiced the exercises daily at home and registered their progression in a diary. In this diary the participants reported per day a) their progression in breath retention at home and b) their mental and physical state. The adherence to the training program was discussed and assessed weekly by the trainers and during the study visits by the medical personnel.

### Assessments

Primary safety assessments included vital signs, physical examination, electrocardiogram, haematology and chemistry at baseline, 4, 8, and 24 weeks and upon indication, and recording of adverse events (AEs) and serious adverse events (SAEs). The number and severity of adverse events was used to assess safety, other safety assessments, such as measuring blood pressure, heart rate, body temperature and physical exam, were done to detect potential adverse events that were not reported by the participants. Secondary endpoints were the change in serum hs-CRP (mg/L) levels between baseline and week 8 of the intervention/control period as a quick response measure, and erythrocyte sedimentation rate (ESR, mm/hr) and serum calprotectin levels[8] (measured by ELISA). Exploratory end-points were disease activity measured by ASDAS-CRP[6] and BASDAI[9], quality of life measured by the short-form 36 (SF-36)[10] and the EuroQol-5D (EQ-5D)[11] and depressive symptoms measured by the Hospital Anxiety Depression Score (HADS)[12]. All questionnaires were self-administered and used and scored according to the test manuals.

### Statistical analysis

Because of the PoC design with safety as primary outcome, sample size could not be calculated and was estimated based on previous work[2]. All patients are included in the analysis of the primary outcome. For the primary outcome, safety, Mann-Whitney U tests were performed. For the secondary and exploratory outcome, a per protocol analysis was performed in which only patients that completed the intervention period were included. For the analyses of the intervention period, the intervention periods of the early and late intervention group were combined. The control group consisted of the first 8 weeks of the late intervention group. Secondary and exploratory outcomes were primarily assessed within groups over the 8-week intervention/control period using Wilcoxon signed rank tests comparing baseline and week 8. Because of the proof-of-concept nature of this trial, we did not adjust for multiple testing. A p value of <0.05 was considered statistically significant.

### Patient and public involvement

This intervention has received extensive media attention in the past years, resulting in patients with various conditions practicing this intervention. However there was little known about safety and putative immunomodulatory effects. The importance of a research project that would increase insight into these matters was highly appreciated by patients as mentioned in patient panels. To properly set the training program for patients with axSpA, 3 patient experts were consulted. As their strong preference was that all patients would receive the intervention, the study was designed as a one-way crossover open-label randomized (within-group) controlled trial. The results will be disseminated to study participants by personal communication and press release.

## Results

**Fig 1:**
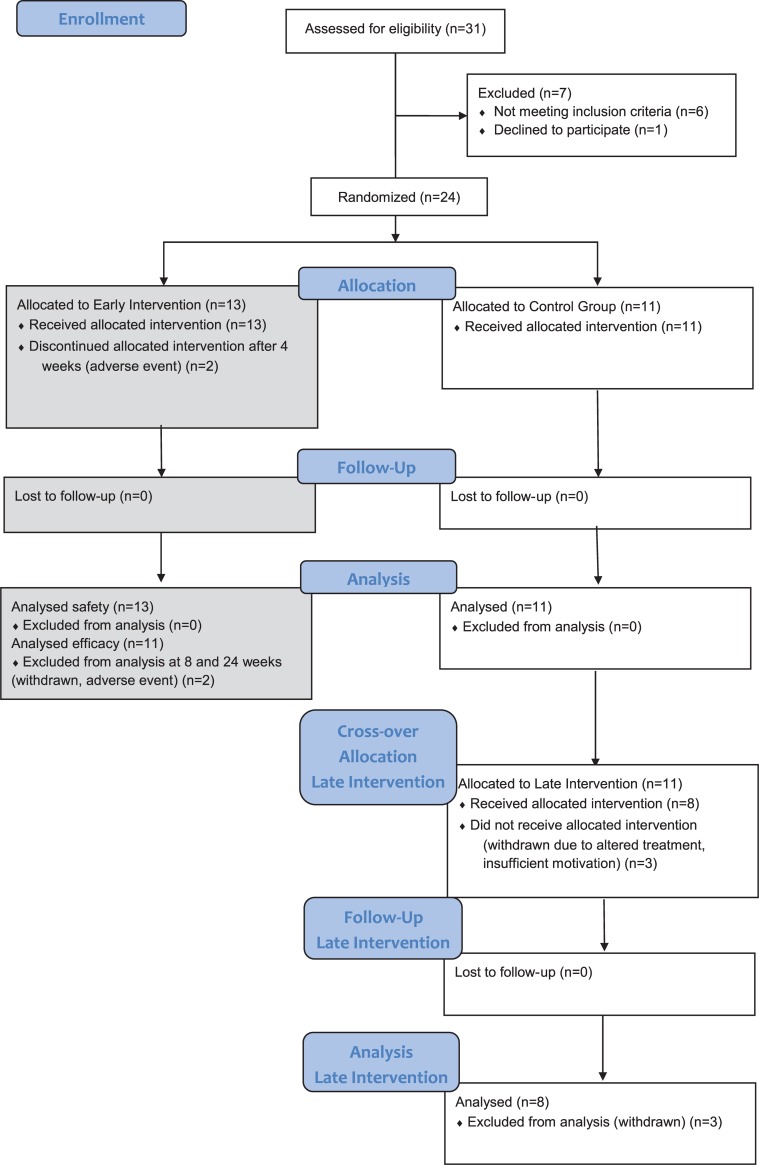
Flow-chart and patients disposition.

Thirty-one patients were screened and 24 patients were randomised to either the early (n = 13) or late intervention (n = 11) group (Fig 1). There were no statistical differences between both groups in demographics, baseline characteristics and concomitant medication (Table 1). Two patients in the early intervention group discontinued after 4 weeks of training: 1 because of an adverse event (AE) and 1 because of a change in medication for a persisting arthritis. In the control group, 3 patients discontinued after the control period (week 8). Two because of insufficient motivation for the intervention, 1 was lost to follow-up. In total 21 participants started with the intervention, of whom 19 completed the intervention period. All 11 patients in the late intervention group completed the control period.

**Table 1:** Demographics, characteristics and medication use at baseline.

	Total study population (n = 24)	Early intervention group (n = 13)	Late intervention group (n = 11)
Male sex, n(%)	15 (62.5)	8 (61.5)	7 (63.6)
Age, years	35.00 ± 7.31	35.46 ±8.29	34.45 ± 6.30
BMI, kg/m^2^	23.22 ± 6.39	24.46 ±3.22	21.44 ±9.25
HLA-B27 positive, n(%)^#^	16/21 (76.1)	11/12 (91.7)	5/9 (55.5)
Hs-CRP at baseline, mg/L, median (IQR)	8.3 (5.4–16.4)	7.9 (6.3–16)	8.7 (5.2–17.2)
ASDAS-CRP	3.02 ± 0.89	3.10 ±0.97	2.93 ± 0.81
Fulfill mNY, n(%)	16 (66.7)	9 (69.2)	7 (63.6)
IBP, n(%)	23 (95.8)	13 (100)	10 (90.9)
Psoriasis, n(%)	1 (4.2)	1 (7.7)	0 (0)
IBD, n(%)	2 (8.3)	1 (7.7)	1 (9.1)
Enthesitis, n(%)	5 (20.8)	4 (30.8)	1 (9.1)
Arthritis, n(%)	9 (37.5)	5 (38.5)	4 (36.4)
Dactylitis, n(%)	1 (4.2)	1 (7.7)	0 (0)
Uveitis, n(%)	6 (25.0)	5 (38.5)	1 (9.1)
Family history positive for SpA, n(%)	8 (33.3)	6 (46.2)	2 (18.2)
Good response to NSAIDs, n(%)	22 (91.7)	11 (84.6)	11 (100)
Elevated hs-CRP, n(%)	24 (100)	13 (100)	11 (100)
NSAIDs, n(%)	22 (91.7)	11 (84.6)	11 (100)
Anti-TNFα, n(%)	3 (12.5)	2 (15.4)	1 (9.1)
Anti-IL17, n(%)	2 (8.3)	1 (7.7)	1 (9.1)
cDMARDs, n(%)	2 (8.3)	2 (15.4)	0 (0)
Corticosteroids, n(%)	1 (4.2)	1 (7.7)	0 (0)
General medication, n(%)	9 (37.5)	5 (38.5)	4 (36.4)

Except where indicated otherwise, values are mean ± SD

^#^HLA-B27 status is missing in 3 patients

IBP inflammatory back pain, IBD inflammatory bowel disease, mNY criteria modified New York criteria, cDMARDs conventional disease modifying anti-rheumatic drugs, NSAIDs non-steroidal anti-inflammatory drugs

### Safety

No serious adverse events occurred during the intervention period. During the intervention period, 10 participants in the intervention group reported a total of 12 AEs (2 participants reported 2 AEs), and 7 participants of the control group reported a total of 9 AEs (2 participants reported 2 AEs)(p = 0.268). The most common AE was common cold (4 vs. 2, respectively, Table 2). Three AEs during the intervention period had a moderate severity (1 in the intervention group and 2 in the control group). During the follow-up period (n = 19), there was one SAE of a participant who experienced a hypertensive crisis 8 weeks after end of the intervention and was hospitalised for one night. Upon further examination by a ophthalmologist there were signs of longstanding disease, therefore this SAE was judged ‘unrelated’ to the study procedures and did not lead to discontinuation of the study.

**Table 2:** Adverse events during the intervention period, control period and follow-up period in absolute numbers and percentages.

	Intervention period (n = 24)	Control period (n = 11)	Follow-up period (n = 19)
Serious adverse event, n(%)	0	0	1(5.3)
≥1 adverse event, n(%)	12(57.1)	9(81.2)	7(36.8)
Anterior uveitis, n(%)	0	1(9.1)*	1(5.3)*
Headache, n(%)	1(4.8)*	0	0
Common cold, n(%)	4(19.0)	2(18.2)	3(15.8)
Other infectious diseases, n(%)	2(9.5)	2(18.2)	1(5.3)
Other adverse events, n(%)	5(23.8)	4(36.4)**	2(10.5)

*Adverse events with moderate severity

**One out of four had a moderate severity

### Secondary endpoints

All secondary endpoints are listed in Table 3. In the intervention group, ESR significantly declined over time: from 16[9–26.5] mm/hr at baseline to 9[5–23] mm/hr at week 8, p = 0.040, while it did not in the control group: from 14[8.3–27.3] mm/hr to 16[5–37] mm/hr, p = 0.406). Hs-CRP did not significantly change within both groups during the 8-week intervention period (intervention group: from median [IQR] 10.2[6.5–17.1] mg/L to 6[3.9–15.6] mg/L, p = 0.103, control group: from 8.7[5.2–17.2] mg/L to 13.2[7.9–20.1] mg/L, p = 0.286). Calprotectin tended to decline in the intervention group (from 2295[1648–4923] pg/mL to 2165[953–3734] pg/mL, p = 0.064), whereas it remained stable in the control group (2115[1300–2571] pg/mL to 2279[1770–4989 pg/mL], p = 0.307).

**Table 3:** Analysis of inflammatory markers in peripheral blood, ASDAS, BASDAI, SF-36, EQ-5D, EQ-5D VAS and HADS during the 8-week intervention period.

	Intervention group			Intra-group p value^#^	Control group			Intra-group p value^#^
	Baseline (n = 21)	4w (n = 21)	8w (n = 19)		Baseline (n = 11)	4w (n = 11)	8w (n = 11)	
hs-CRP (mg/l)	10.2 (6.5–17.1)	8.9 (3.5–13.1)	6 (3.9–15.6)	.103	8.7 (5.2–17.2)	11 (8.4–20.2)	13.2 (7.9–20.1)	.286
ESR (mm/hr)	16 (9–26.5)	10 (6.5–31.5)	9 (5–23)	.040*	14 (8.3–27.3)	12 (8–34)	16 (5–37)	.406
Calprotectin (pg/ml)	2295 (1648–4923)	2245 (1273–3245)	2165 (953–3734)	.064	2115 (1300–2571)	2086 (1221–3527)	2279 (1770–4989)	.182
ASDAS-CRP	3.1 (2.5–3.6)	2.5 (1.9–3.2)	2.3 (1.7–3.2)	.044*	2.9 (2.3–3.6)	3.4 (3–3.6)	3.1 (2.7–3.6)	.213
BASDAI	4.5 (3–5.9)	3.3 (2.1–5.8)	2.6 (1.4–3.5)	.012*	3.2 (2.8–5.5)	5.5 (3–6.2)	4.3 (2.5–5.7)	.755
SF-36 PCS	44.8 (36.0–48.8)	44.6 (39.9–47.4)	49.3 (40.7–54.7)	.004*	41.8 (34.1–49.1)	42.1 (35.9–46.1)	42.8 (33.5–48.0)	.859
SF-36 MCS	45.5 (39.9–53.5)	50.4 (44.3–54.6)	53.5 (46.8–56.7)	.004*	42.0 (38.1–50.4)	45.0 (37.2–53.9)	42.6 (40.0–52.5)	.859
EQ-5D	.81 (.65-.84)	.81 (.67-.92)	0.84 (.81–1.00)	.102	.81 (.69-.84)	.81 (.25-.81)	.81 (.65-.84)	.933
EQ-5D VAS	66.5 (59.3–74.0)	70.0 (55.5–76.0)	75.5 (70.0–87.0)	.090	67.5 (48.3–76.3)	60.0 (45.0–72.5)	66.5 (62.0–75.5)	.674
HADS-Anxiety	5.0 (2.5–7.5)	4.0 (2.5–6.0)	4.0 (.5–6.0)	.369	5.0 (3.0–7.0)	5.0 (4.0–6.0)	3.0 (1.0–5.0)	.138
HADS-Depression	3(1–6)	3(1–6)	2(1–6)	.508	3(2–6)	4(1–5)	1(1–4)	.137

Values are presented as median (interquartile range).

*: *P* < .05

^#^Wilcoxon signed rank test

### Exploratory endpoints

All exploratory endpoints are listed in Table 3. There was a significant improvement in the median ASDAS-CRP during the 8-week period in the intervention group (p = 0.044), but not in the control group (p = 0.213). Median BASDAI declined significantly in the intervention group (p = 0.012) but not in the control group (p = 0.755). The physical component score (PCS) of the SF-36 increased significantly during the intervention period (p = 0.004) but not in the control group (p = 0.859).The MCS of the SF-36 increased significantly in the intervention group (p = 0.004) but not in the control group (p = 0.859).The EQ-5D did not significantly change in the intervention group (p = 0.102) nor in the control group (p = 0.933).A similar effect was observed for the EQ-5D VAS (p = 0.090 and p = 0.674 in the intervention and control groups, respectively. There was no significant effect on the HADS anxiety and depression scales within groups.

The main burden of the intervention assessed by patients themselves was the commuting time to the academic center for group training and follow-up visits. The time spent on group and personal trainings were assessed as purposeful and the burden of cold exposure was transient. The participation during the training sessions was judged as high by the trainers. The adherence to the exercises at home was discussed in the group sessions and reviewed in the diaries, and was also judged as high.

## Discussion

In the present trial we show that the add-on training program involving breathing exercises, cold exposure, and meditation can safely be applied in patients with axial spondyloarthritis, a prototype chronic inflammatory disease.

We observed no differences in the number and severity of adverse events in the intervention group compared to the control group. There was a significant decrease in ESR levels over time in the intervention group but not in the control group. This is in line with previous work showing that the immune response can be modulated through the add-on training program in healthy participants during experimental endotoxemia[2]. The intervention also tended to result in decreased serum calprotectin levels, a validated disease activity biomarker in axSpA[8], although this did not reach a statistical significance. Various measures of disease activity (ASDAS-CRP, BASDAI) and quality of life (SF-36) improved following the intervention. The effects observed in the intervention group are unlikely to be due to regression to the mean as there were no effects in the control group. Therefore, our results are indicative that voluntary modulation of the immune response may not only be possible in acute inflammatory response due to microbial stimulation but also in chronic inflammation related to immune-mediated inflammatory diseases.

This study has several limitations. 1) As this study was a proof-of-concept trial the sample size was small and not powered to investigate efficacy. However, despite this small sample size, significant changes in the secondary and exploratory outcomes were found. Larger follow-up studies are required to formally assess clinical efficacy.

2) The unblinded design of the study renders our results susceptible for a placebo effect. As the studied intervention is not suitable for a genuine placebo treatment, we chose hs-CRP, ESR and calprotectin as a secondary biologic endpoint that does not suffer from subjective influences or variability in the measurements. Furthermore, we used a delayed intervention group to assess the impact of regression to the mean. With this design we neutralized the aforementioned components of placebo effect. Moreover, the data obtained did not suggest an effect of regression to the mean since we observed changes in the intervention but not in the control group.

3) Our study design was not aimed at and therefore does not allow us to decipher the mechanism of action of this intervention. The mechanism of action behind the add-on training program remains to be unraveled. Kox et al clearly showed the biological impact of the training program on the innate immune response[2]. Firstly it is unknown whether it is necessary to practice all three components of the training program, or whether the observed effect in immune response is attained by one of the components. Secondly it is unknown what the minimally required intensity or duration of the training program should be to see similar results. Future research should address these questions.

4) The adherence to the training program at home was not formally checked, although discussed weekly during the group sessions and study visits. The adherence to the group sessions was high as on average the participants missed 1.5 out of 12 group sessions, due to holidays, other obligations or sickness.

5) Although not a major part of the add-on training program, the strength exercises (performed in conjunction with the breathing exercises) might play a role in the improvements we found in the participants, since exercise is a pivotal part of the treatment of axial spondyloarthritis.

In conclusion, the present study demonstrates that the add-on training program used in this study can safely be applied in patients with axial spondyloarthritis and potentially modulates inflammatory response. These findings warrant further clinical assessment of this novel therapeutic approach. Future research should include a larger sample size to formally evaluate clinical efficacy and should by focussed on further elucidation of the mechanism of action of the combined and/or individual components of the training program.

## Supporting information

S1 CONSORT 2010 Checklist (PDF)

S1 Fig Study design. (TIF)

S1 File SPSS database. (SAV)
