# Supplementary figures and images for: An add-on training program involving breathing exercises, cold exposure, and meditation attenuates inflammation and disease activity in axial spondyloarthritis – A proof of concept trial
Source: PLoS One. 2019 Dec 2;14(12):e0225749. doi: 10.1371/journal.pone.0225749 (PMC6886760; doi:10.1371/journal.pone.0225749)

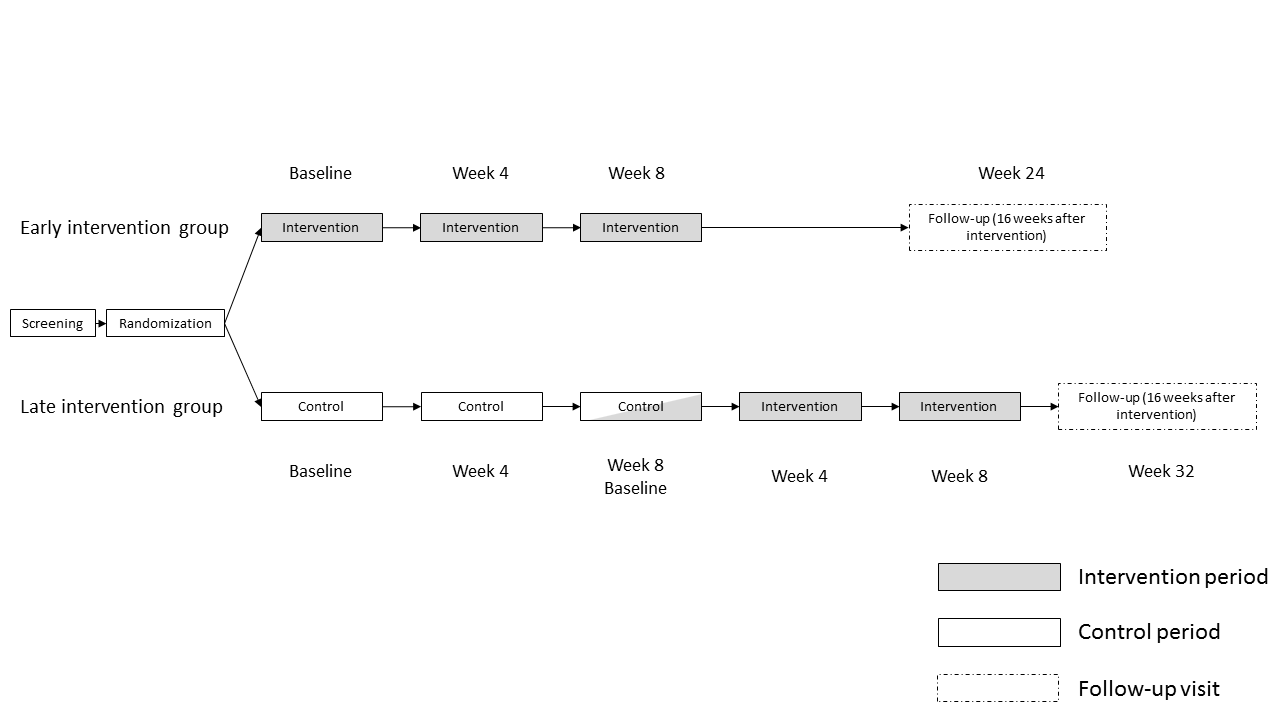

Supplement: S1 Fig — (TIF) [file pone.0225749.s002.tif]
